# Selected Aspects of Reproductive Behavior of American Mink (*Neovison vison*) under Artificial Conditions

**DOI:** 10.3390/ani13213346

**Published:** 2023-10-27

**Authors:** Beata Seremak, Bogumiła Pilarczyk, Aleksandra Wojciechowska, Agnieszka Tomza-Marciniak

**Affiliations:** Department of Animal Reproduction Biotechnology and Environmental Hygiene, Faculty of Biotechnology and Animal Husbandry, West Pomeranian University of Technology in Szczecin, Janickiego 29, 71-270 Szczecin, Poland; beata.seremak@zut.edu.pl (B.S.); bogumila.pilarczyk@zut.edu.pl (B.P.); aleksandra.maria.wojciechowska@gmail.com (A.W.)

**Keywords:** American mink, reproduction, reproductive behavior, mating

## Abstract

**Simple Summary:**

The aim of this study was to evaluate the mating behavior of the male American mink, with regard to its duration and any potential differences with regard to time of day, mating period, number of mating attempts, and time spent with the female. Individual differences in copulation duration were observed among the studied males. However, the length of copulation time generally increased over the successive days of the breeding season. It was also observed that a longer time spent with the female to initiate copulation, and behaviors such as rubbing or sniffing the female, were associated with a shorter copulation time.

**Abstract:**

Observation and behavioral analysis of animals can be one of the factors taken into consideration when assessing the welfare of animals kept in artificially created breeding conditions. Disturbed welfare and prolonged exposure to stress can lead to the emergence of abnormal behaviors, including reproductive issues. The aim of this study was to examine the mating behavior of male American mink, with regard to the duration of mating and any potential differences with regard to time of day, mating period, number of mating attempts, and time spent with the female. The study material consisted of 12 one-year-old male American minks with pearl coloring and 60 two-year-old females. Observations were conducted using images captured using externally mounted cameras. The video material capturing the activity of males and females during the mating period was analyzed using the Behawior program. The obtained results indicate the presence of individual differences in copulation duration. It was observed that the copulation time increases with successive days of the breeding season and with number of copulations. It was also observed that a longer time spent with the female to initiate copulation, and behaviors such as rubbing or sniffing the female, were associated with a shorter copulation time. The above-mentioned studies provide specific guidelines regarding the management of matings on the farm, demonstrating the feasibility of conducting matings both in the hours before and after noon.

## 1. Introduction

With the intensification of animal production, numerous problems have emerged regarding ensuring proper welfare for animals kept in farm conditions. Animal welfare is a system intended to provide animals with the best living conditions that meet their behavioral needs, and one that guarantees a high level of professional care. Commissions established within the European Union aim to improve the quality of animal life through the issuance of appropriate regulations and monitoring their implementation. Modern farming, therefore, must meet many imposed requirements. As a result, there is great interest in the scientific study of animal behavior, as its analysis provides direct information about welfare. Monitoring and analyzing animal behavior are thus important criteria for determining welfare. They are a non-invasive method that provides important information regarding the physical and psychological health of animals [1]. Artificially created breeding conditions should allow for the expression of natural behaviors specific to each species. Behavioral patterns form complex systems referred to as functionally coherent sets of behavior norms controlled by environmental stimuli, motivational mechanisms, and sensory-motor processes [2].

Familiarity with the behavioral patterns characteristic of a given species allows for the detection of deviations from the norm, which are defined as a series of motor activities, vocalizations, and social interactions that occur when animal welfare is compromised [3,4]. Behavioral systems are quite similar to physiological systems and do not function completely independently of each other; many of them are directly interconnected [5,6]. Sexual behaviors, for example, can be an example. Prolonged exposure to stress causes a wide range of physiological changes in the body, leading to adverse effects on its functioning, including reproductive issues [7]. The impact of stress on reproduction has been observed in both males and females. The response to a stressor can disrupt mammalian reproduction by delaying oocyte development, blocking estrus, and disturbing hormonal balance. In the case of males, stress has a negative impact on androgen production and the process of spermatogenesis [8].

The American mink is considered a monoetral animal, because in both the wild and in captive conditions, the species engages in reproduction once a year [9,10]. The onset of the breeding season in mink is influenced by both the availability of food in the environment and the change in length of daylight. The lengthening day stimulates the hypothalamus to produce gonadotropin-releasing hormone, which in turn activates the pituitary gland to synthesize gonadotropic hormones that regulate reproductive processes [11,12]. The estrous phase is continuous, lasting from 7 to 20 days [13], and consists of constant, consecutive cyclic periods of oocyte maturation and ovulation. The length of the cycles has been found to vary, repeating every six days or so [14], or every seven [15], seven to nine [16], seven to ten [17], or even ten days [18]. Some authors [15,16,19] emphasize that up to four of these cycles can occur in mink during the breeding season.

In the Northern Hemisphere, the breeding season of the American mink begins when the day becomes roughly two hours longer than the eight-hour winter day [20,21]. However, the duration of the breeding season demonstrates some degree of variation, beginning as early as the end of February, and lasting about four weeks, as noted by Pilbeam et al. [22], Lagerkvist et al. [16], and Gulevich et al. [23]. Fink et al. [24] reports the period to occur for about three weeks in March [13], while Sundqvist et al. [19] indicate that it includes the whole of March, and Travis [25] and Persson [26] found it to extend as far as early April. In wild populations living at different latitudes, this period may shift and start as late as May [27,28,29,30,31,32]. In farm practice, it is common to group the herd into breeding sets. A typical set consists of five sections, each containing eight or six related females, and a sixth section with eight or six unrelated males. The males in the groups are typically related to each other but not to the females [33,34]. In Poland, the breeding season on farms begins in early March. Each day, females are introduced to the males’ cages. Mating behavior in mink, as described by Dallaire and Mason [35], can be preceded by “courtship” of varying duration, resembling a fight or chase around the cage.

The aim of this study was to evaluate the mating behavior of the male American mink, with regard to its duration and any potential differences with regard to time of day, mating period, number of mating attempts, and time spent with the female.

## 2. Materials and Methods

The experiment was conducted on a mink farm located in northern Poland. The animals were fed a standard semi-liquid diet based on chicken and fish, supplemented with minerals and vitamins, and remained in the same environmental conditions throughout the experiment (cage system). The animals were kept in accordance with the European Convention for the Protection of Vertebrate Animals and met the requirements of the Polish Act of 29 June 2007, and the Regulation of the Minister of Agriculture and Rural Development of 10 September 2015, regarding the minimum conditions for keeping farm animal species, which have been in effect since 1 January 2018. The minks were housed individually in standard cages (length/width/height = 90/45/45 cm), and they did not show any behavioral disorders.

According to Polish law, due to the non-invasive nature of the procedures, this study did not require approval from the ethical committee for animal research [36].

The research material consisted of 12 one-year-old male American minks of the pearl color variety, randomly selected, and 60 two-year-old females, also of the pearl variety. Copulation took place in the male’s cage, to which the females were transferred, and its duration was measured from the moment of the introduction of the male’s penis into the female’s reproductive tract to the characteristic bending of the pelvis, separation of the animals, and their distancing from each other. The females were mated according to the following scheme: 1 + 2 + 8 + 9, i.e., mating on the first, second, eighth, and ninth days since the first mating attempt. It is important to note that this does not mean that the first mating occurred on the 6 March, i.e., the first day of the season; it could have been the 7th or 8th. Each female was mated on the day after the first mating (day 2), then again on days eight and nine. Each day, each male mated with two different females twice: once in the morning from 6 a.m. and once again in the afternoon from 12 p.m. Mating was carried out within the breeding set (30 females—5 groups of 6 females each; 6 males—1 group). The females within each group were related to each other. Females were mated with various males from the breeding set. This is common practice on the farm.

If a morning or afternoon mating was not successful, the female was exchanged for another from the same group of related females. This potentially allowed the mating schedule to be maintained by allowing the males to mate with two females per day.

A copulation attempt was considered successful if it lasted more than 10 min. If it was unsuccessful, the female was matched the next day with another male. Copulation was never interrupted while ongoing.

The experiment was made possible by cameras mounted outside the cages, recording and saving the footage. The animals were monitored from dawn to dusk (from 06:00 a.m. to 18:00 p.m.) during the breeding season, which lasted from 6 March to 17 March. In total, 1728 h of recorded footage was reviewed and analyzed. All behaviors of the observed individuals were recorded throughout the duration of the experiment. Data were collected using the serial recording method, which involved documenting all behaviors occurring in a specific group within defined time intervals. This method allowed for recording the duration, order of appearance of specific behaviors, and interactions between individuals. The video material depicting the activity of males and females during the pre-mating period was analyzed using the Behawior program.

To determine the influence of date of mating on the observed reproductive behaviors, the entire mating season was divided into two periods: the first from 6 March to 11 March, and the second from 12 March to 17 March.

The results were analyzed using STATISTICA^®^ (StatSoft Inc., ver. 13.3 StatSoft, Tulsa, OK, USA). The student’s *t*-test was used to verify the influence of time of day and mating period on copulation duration. The strength of the relationship was measured using the Cohen’s d coefficient calculated using the formula: d = 2t/√df, where t is the t-statistic and df is the degrees of freedom. The nonparametric Pearson’s chi-squared test was used to assess the association between the number of mating males in different seasons and time of mating day. The relationship between copulation duration and mating season was analyzed using regression coefficients. The relationship between the time preceding copulation (from introducing the female into the male’s cage to the initiation of copulation) and the copulation time itself was analyzed using Spearman’s rho correlation analysis.

## 3. Results

The mean copulation time and the number of copulations for individual males in the first and second mating periods are given in Table 1 and Table 2.

The mean copulation time was 52 min and 37 s in the first mating period (from 6 March to 11 March) and one hour, four minutes and 32 s in the second period (from 12 March to 17 March).

In both of the analyzed periods, we observed varying average copulation durations among the experimental males. We observed copulations lasting both over an hour and those that were half as long.

To compare the copulation time for each individual within each of two analyzed periods, the independent samples *t*-test was applied (Table 3). The results indicate that the date of mating only had a significant impact on the copulation time for male number 2 (t (18) = −2.24, *p* < 0.05, d = 1.05), for which the second mating period had a significantly longer mean copulation time, i.e., 1:12:47, compared to 00:53:12 for the first period. No statistically significant differences were found for the other individuals.

**Figure 1:**
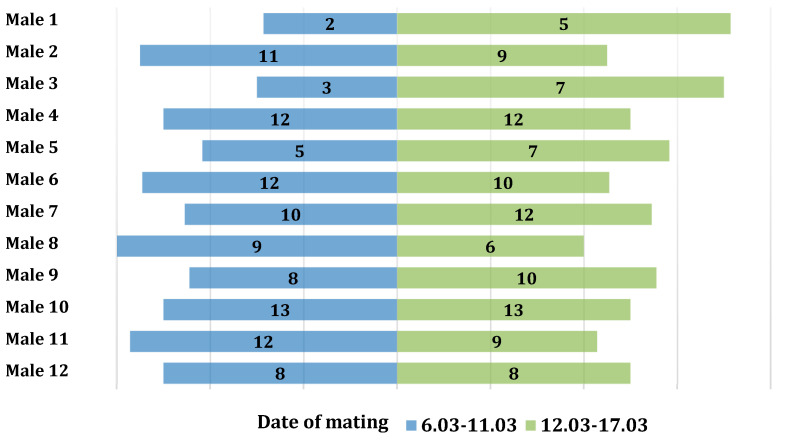
Distribution of the number of copulations for individual males divided into the first and second mating periods.

The association between the number of copulations and the times of day when mating occurred was examined using the Pearson’s chi-squared test. No significant relationship was found between the number of copulations and the mating periods (χ2(11) = 4.99, *p* = 0.932, V = 0.15). In the first period, 104 copulations were observed, while in the second period, 109 copulations were recorded. The number of copulations in the two analyzed periods was symmetrical (Figure 1).

To determine the impact of the time of day on the observed copulation behavior, two time intervals were identified: 6:00 to 12:00 a.m. and 12:00 a.m. to 06:00 p.m. Table 4 and Table 5 present the mean copulation time and the number of copulations for individual males during the morning and afternoon periods.

The copulation times in the examined time intervals were compared using the Student’s *t*-test for independent samples. No statistically significant differences were found (*p* > 0.05) between the mating season and the mean copulation time in males (Table 6). A significant difference was only found in the case of individual number 8 (t (13) = −2.30; *p* < 0.05; d = 1.28), where the mean copulation time in the afternoon was significantly longer than in the morning.

**Figure 2:**
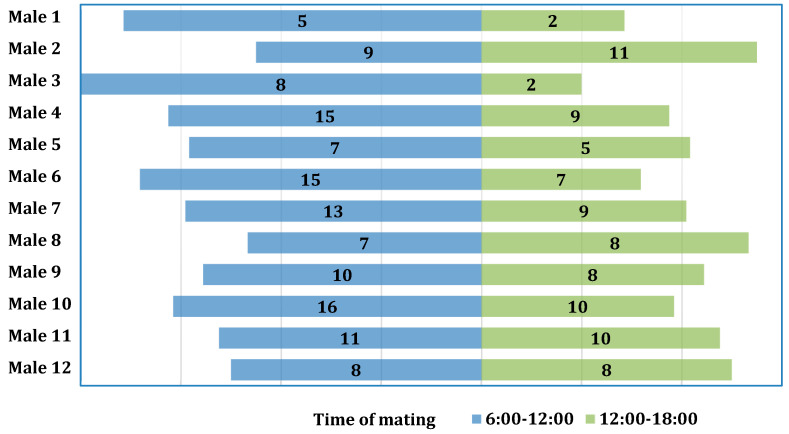
Distribution of the number of copulations for individual males with regard to time of day.

The chi-square test was used to examine the association between the number of copulations and the times of day during which mating occurred, but it was found to be non-significant (χ2(11) = 6.71; *p* = 0.822; V = 0.12) (Figure 2).

The mean copulation time for all males combined was compared across consecutive breeding days. The analysis indicates that the breeding date appears to have a statistically significant effect on the mean copulation time (t (211) = −3.13; *p* < 0.01; d = 0.43), with a longer time observed during the period of 12 February to 17 March (1:04:32) compared to 6 March to 11 March (00:52:37). An analysis was conducted to determine if the mean copulation time varied depending on the day of the breeding season (Table 7). The regression coefficient was found to be statistically significant: F (1,211) = 9.80; *p* < 0.01; R^2^ = 0.04. On each consecutive day, the copulation time increased in mean by 11.42 min ± 3.51 min (t = 3.13; *p* < 0.01). However, the variability in copulation time was only slightly explained by the day-to-day variation (R^2^ = 0.04).

Table 8 presents the mean times and number of copulations for individual days during the mink breeding season. The day with the highest number of copulations was 8 March, followed by 13 March, while the lowest number of copulations was observed on 10 March. The number of copulating males ranged from 7 to 12 without a clear trend in consecutive days. The number of copulations also varied among different days, and no relationship related to the breeding date was observed.

**Figure 3:**
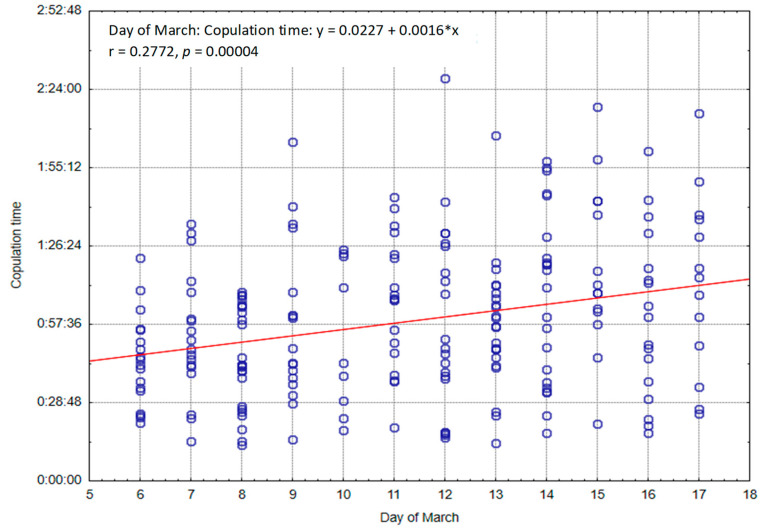
Length of all copulations (h:m:s) with regard to day in March.

Figure 3 shows the lengths of all copulations performed by individual males during the period from March 6 to 17, together with the observed trend. A significant (*p* < 0.05) positive correlation can be seen between the duration of copulation and consecutive days.

It was shown that the length of copulation time was positively correlated with the number of copulations. However, this relationship was only statistically significant in the case of three males (Table 9).

The relationship between the length of copulation and the time from the entry of the female into the cage was analyzed using Spearman’s correlation coefficient (Table 10). The results show that, in general, the length of copulation was significantly related to the length of time from the entry of the female into the cage until copulation (ρ = −0.27; *p* < 0.001).

The relationship was negative, i.e., a longer period of contact with the female before copulation was associated with shorter copulation. Males that spent more time sniffing the female and rubbing against her were characterized by shorter copulation times. A strong relationship between copulation time and the length of time before copulation was noted in males 5, 8, and 9.

The relationship between the copulation time and the length of individual reproductive behavior was analyzed using correlation analysis (Pearson’s r; Table 11). The results showed that the copulation time of males was significantly related to the time spent sniffing the female (r = −0.24; *p* < 0.01) and rubbing against her (r = −0.65; *p* < 0.05). It was shown that males who spent more time sniffing and rubbing the female had shorter copulation times.

## 4. Discussion

In addition to our findings related to the act of copulation itself, which themselves may represent valuable information for farm owners organizing the mating season, they also reveal a diverse repertoire of mink reproductive behavior (Table 11). Among the observed behaviors were sniffing the female, race, and observation. Reports of observed reproductive behaviors in wild mink corresponded to those observed in our study. Before engaging in copulation, male American mink sniff the vicinity of the anus, vulva, and back of the female. Based on this, males can determine the sex of the animal and the phase of the female’s reproductive cycle [37]. The male was observed to demonstrate varying levels of interest in the female, resulting in varying lengths of time between the entry of the female to the start of copulation, as shown in Table 9. The precopulatory behavior focused mainly on mutual sniffing, rubbing, and the minks chasing each other around the cage. These observations are in line with those of wild animals noted by other authors, which may indicate adaptation and acclimatization to farm conditions. Similar to the behaviors observed in our own study, observations on mink caught from the wild indicate that copulation is often preceded by ”courtship” behaviors resembling fighting or chasing around the cage [30,35].

Similar courtship behaviors preceding mating were also observed by Lodé [38] during a two-year observation of captive beech martens (*Martes foina*) released into enclosures. Poole [39] also observed such behaviors in polecats (*Mustela putorius*).

The male was observed to rub against the female and also against the cage; this likely served as a landmarking with secretions of the perianal glands. Such behavior has been observed in both wild mink and other mammalian species [30,40]. Chemosensory cues play a crucial role in the reproductive process of monoestrous females [41,42,43,44].

The study analyzed the relationship between selected reproductive behavior and copulation time (Table 10). It was found that a longer period of time spent on specific reproductive rituals prior to copulation was associated with a shorter copulatory act. The conducted research also indicated that some females adopted defensive postures, trying to avoid contact with the male or attempting to escape his grip during copulation. Others showed interest in the male and a willingness to mate. The observed behaviors of females and the analyzed factors (date of mating and time of day) influenced the varied duration of copulation. Similar observations were made by Hansson [31], who noted the shortest copulations at the beginning of the breeding season, with the mean duration increasing as the mating season progressed. The mean copulation time in the cited studies before 10 March was 49 min, which increased to 114 min after 26 March. Elofson et al. [45] observed a gradual increase in female acceptance of males in the following days of the breeding season, as well as an extension of the mean copulation duration from 32 min on 7 March to 102 min on 22 March. The authors reported that the length of copulation increased on mean by 4.2 min on consecutive days. Fleming [46] also noted a gradual increase in copulation duration in the following days of the breeding season. During the first period of the breeding season, the copulation time increased by a mean of about 4 min each day, while in the subsequent period, this time increased by a mean of 3 min per day. Our findings indicate that the mean copulation time at the beginning of the mating season (from 6 to 11 March) was about 12 min shorter than that of the second mating date. The values obtained in our own research, presented in Table 4 and Figure 3, coincide with the results mentioned above by the authors who reported an increase in the mean copulation time on successive days of the breeding season. As Fleming [46] suggests, shorter copulations were observed at the beginning of the mating season, and the extension of copulation duration in the following days of the mating season on the farm may be due to changes occurring in animal behavior, along with the progression of the reproductive cycle and an increase in their activity.

According to Venge [47], females are most receptive to male courtship at the end of the mating season. Females that were first mated after 20 March usually immediately accepted the male. Additionally, as reported by Johansson and Venge [48] and Shackelford [49], approximately 90% of offspring are conceived during the mating in the last ovulation of the breeding season.

## 5. Conclusions

Individual differences in copulation duration were observed among the studied males, with some experiencing longer copulation periods while others had shorter ones. It was observed that the copulation time increased in the successive days of the breeding season and with number of copulations. It was also observed that a longer time spent with the female to initiate copulation, and behaviors such as rubbing or sniffing the female, were associated with a shorter copulation time.

## Figures and Tables

**Table 1:** Mean copulation time and number of copulations for individual males in the first mating period.

First Mating Period (6 March–11 March)					
Male Number	Number of Copulations	Mean Copulation Time	Standard Error SE	Min.	Max.
		[h:m:s]		[h:m:s]	[h:m:s]
1	2	00:50:45	00:19:21	00:31:23	01:10:07
2	11	00:53:12	00:06:09	00:21:20	01:23:26
3	3	00:57:25	00:19:50	00:26:30	01:34:24
4	12	00:32:46	00:03:40	00:15:11	00:59:19
5	5	00:47:18	00:08:38	00:19:43	01:06:50
6	12	00:52:13	00:08:12	00:14:24	02:04:42
7	10	00:47:00	00:07:33	00:22:36	01:44:25
8	9	00:43:09	00:06:27	00:14:37	01:11:00
9	8	01:08:32	00:07:58	00:40:14	01:40:10
10	13	00:46:22	00:04:32	00:13:11	01:09:28
11	12	01:06:57	00:06:52	00:24:48	01:33:44
12	8	01:14:31	00:09:16	00:25:30	01:40:57
Total	105	00:52:37			

**Table 2:** Mean copulation time and number of copulations for individual males in the second mating period.

Second Mating Period (12 March–17 March)					
Male Number	Number of Copulations	Mean Copulation Time	Standard Error SE	Min.	Max.
		[h:m:s]		[h:m:s]	[h:m:s]
1	5	01:13:08	00:21:44	00:24:00	02:15:20
2	9	01:12:47	00:06:04	00:37:34	01:42:53
3	7	01:00:27	00:10:24	00:17:50	01:31:04
4	12	00:30:26	00:04:17	00:13:46	00:49:40
5	7	01:28:18	00:14:21	00:34:12	02:28:13
6	10	00:59:38	00:09:08	00:22:49	01:58:18
7	12	01:08:41	00:09:07	00:20:15	02:07:05
8	6	01:04:42	00:13:50	00:21:06	01:55:08
9	10	01:07:03	00:07:53	00:41:40	01:57:38
10	13	00:54:51	00:07:18	00:13:55	01:36:13
11	9	01:24:02	00:07:35	00:48:30	02:01:25
12	8	01:14:15	00:11:32	00:42:18	02:17:41
Total	108	01:04:32			

**Table 3:** The relationship between copulation time and mating period for individual minks (independent samples *t*-test).

Changes in Mean Copulation Time Based on the Mating Period				
Male Number	*t*	*df*	*p*	*d*
1	−0.59	5	0.580	0.53
2	−2.24	18	0.038	1.05
3	−0.15	8	0.884	0.11
4	0.41	22	0.685	0.18
5	−2.20	10	0.053	1.39
6	−0.60	20	0.553	0.27
7	−1.78	20	0.090	0.80
8	−1.58	13	0.139	0.87
9	0.13	16	0.897	0.07
10	−0.99	24	0.334	0.40
11	−1.66	19	0.114	0.76
12	0.02	14	0.986	0.01

*t*—student’s *t*-test, *df*—degrees of freedom, *p*—statistical significance level, *d*—Cohen’s effect.

**Table 4:** Mean copulation time and number of copulations for individual males in the first time interval (6.00 a.m.–12.00 p.m.).

First Time Interval 6.00–12.00 a.m.					
Male Number	Number of Copulations	Mean Copulation Time	Standard Error SE	Min	Max
		[h:m:s]		[h:m:s]	[h:m:s]
1	5	01:05:47	00:18:29	00:31:23	02:15:20
2	9	00:58:27	00:04:30	00:38:01	01:16:40
3	8	01:00:57	00:08:42	00:26:30	01:34:24
4	15	00:32:09	00:03:13	00:13:46	00:48:44
5	7	01:12:04	00:17:03	00:19:43	02:28:13
6	15	00:55:54	00:07:46	00:23:28	02:04:42
7	13	00:59:33	00:09:10	00:22:36	02:07:05
8	7	00:36:37	00:06:18	00:21:06	01:09:14
9	10	01:07:07	00:06:01	00:40:14	01:40:10
10	16	00:50:14	00:04:55	00:13:11	01:12:09
11	11	01:19:50	00:08:39	00:24:48	02:01:25
12	8	01:15:13	00:12:46	00:25:30	02:17:41
Total	124	00:57:41		--	

**Table 5:** Mean copulation time and number of copulations for individual males in the second time interval.

Second Time Interval 12.00 a.m.–06.00 p.m.					
Male Number	Number of Copulations	Mean Copulation Time [h:m:s]	Standard Error SE	Min [h:m:s]	Max [h:m:s]
1	2	01:09:08	00:45:08	00:24:00	01:54:16
2	11	01:04:55	00:08:00	00:21:20	01:42:53
3	2	00:53:53	00:36:03	00:17:50	01:29:56
4	9	00:30:42	00:05:21	00:15:11	00:59:19
5	5	01:10:02	00:11:52	00:42:56	01:50:12
6	7	00:54:56	00:09:47	00:14:24	01:18:13
7	9	00:57:47	00:08:48	00:20:15	01:42:34
8	8	01:05:02	00:10:07	00:14:37	01:55:08
9	8	01:08:27	00:10:16	00:41:40	01:57:38
10	10	00:51:12	00:08:18	00:13:55	01:36:13
11	10	01:08:10	00:05:43	00:39:46	01:33:44
12	8	01:13:32	00:07:28	00:45:30	01:45:06
Total	89	01:00:01	--	--	--

**Table 6:** The relationship between copulation time and examined time intervals for individual males (Student’s *t*-test for independent samples).

Change in Mean Copulation Time with Regard to Time Interval				
Male Number	*t*	*df*	*p*	*d*
1	−0.09	5	0.935	0.08
2	−0.66	18	0.517	0.31
3	0.31	8	0.767	0.22
4	0.25	22	0.807	0.11
5	0.09	10	0.931	0.06
6	0.07	20	0.943	0.03
7	0.13	20	0.896	0.06
8	−2.30	13	0.039	1.28
9	−0.12	16	0.908	0.06
10	−0.11	24	0.917	0.04
11	1.10	19	0.285	0.51
12	0.11	14	0.911	0.06

*t*—student’s *t*-test, *df*—degree of freedom, *p*—level of statistical significance, *d*—Cohen’s effect size.

**Table 7:** Results of linear regression analysis for the variability of copulation time across consecutive days.

Parameter	*B*	*SE*	*β*	*t*	*p*
	40.70	6.05		6.73	0.000
Day	11.42	3.51	0.21	3.13	0.002

*B*—unstandardized coefficient, *SE*—standard error, *β*—standardized coefficient, *t*—student’s *t*-test, *p*—level of statistical significance.

**Table 8:** Mean time and number of copulations for the entire group of males with regard to individual days.

Day	Number of Copulations	Number of Males Mating	Mean Copulation Time	Standard Error SE	Min	Max
			[h:m:s]		[h:m:s]	[h:m:s]
6 March	19	11	00:44:31	00:04:03	00:21:20	01:31:20
7 March	17	10	00:51:56	00:05:59	00:14:37	01:34:24
8 March	25	11	00:45:09	00:03:41	00:13:11	01:09:37
9 March	17	11	00:57:50	00:07:13	00:15:11	02:04:42
10 March	9	8	00:52:53	00:09:12	00:18:39	01:25:02
11 March	18	10	01:07:05	00:05:36	00:19:43	01:44:25
12 March	19	11	00:58:24	00:07:59	00:15:46	02:28:13
13 March	24	12	00:56:30	00:05:14	00:13:46	02:07:05
14 March	21	12	01:06:31	00:06:51	00:17:30	01:57:38
15 March	14	9	01:17:49	00:08:12	00:21:06	02:17:41
16 March	17	9	00:59:39	00:07:11	00:17:28	02:01:25
17 March	13	7	01:17:13	00:08:48	00:24:36	02:15:20

**Table 9:** Correlation between copulation time and number of copulations.

Male	Number of Copulations	R Value	*p* Value
1	7	0.43	0.337
2	20	0.48	0.032
3	10	0.01	0.987
4	23	0.01	0.974
5	12	0.60	0.039
6	22	0.26	0.236
7	22	0.05	0.844
8	15	0.35	0.201
9	18	0.29	0.247
10	27	0.21	0.289
11	21	0.60	0.004
12	18	0.13	0.616

**Table 10:** Correlation between time before copulation (after introducing the female into the cage) and copulation time.

Male	Number of Copulations	Mean Time before Copulation	Mean Copulation Time	Spearman’s Correlation
		[h:m:s]	[h:m:s]	R Value
1	7	00:14:24	01:06:44	0.00
2	20	00:21:46	01:02:01	−0.40
3	10	00:23:52	00:59:33	−0.18
4	23	00:10:13	00:31:37	−0.20
5	12	00:33:12	01:11:14	−0.67 *
6	22	00:43:52	00:55:36	0.04
7	22	00:24:25	00:58:50	−0.41
8	15	00:04:50	00:51:47	−0.55 *
9	18	00:02:41	01:07:43	−0.48 *
10	27	00:11:27	00:50:37	0.05
11	21	00:06:34	01:14:17	0.41
12	18	00:27:50	01:14:23	−0.53
Total	17.92	00:18:45	01:00:23	−0.27 **

* *p* < 0.05; ** *p* < 0.001.

**Table 11:** Correlation between individual types of male behavior and copulation time.

Spearman Correlation, r Value	
Types of Behavior	Copulation Time
Rest	−0.14
Race	−0.10
Sniffing the female	−0.24 **
Rubbing	−0.65 *
Observation	−0.06
Care	0.02
Game	−0.09

* *p* < 0.05; ** *p* < 0.001.

## Data Availability

The data presented in this study are available on request from the corresponding author.
